# Sectoral output, energy use, and CO_2_ emission in middle-income countries

**DOI:** 10.1007/s11356-017-8599-z

**Published:** 2017-03-01

**Authors:** Kazi Sohag, Md Al Mamun, Gazi Salah Uddin, Ali M. Ahmed

**Affiliations:** 1 Institute of Climate Change, Universiti Kebangsaan Malaysia, Bangi, Selangor Malaysia; 2 Department of Economics and Finance, La Trobe University, Melbourne, VIC 3086 Australia; 3 East West University, Dhaka, 1212 Bangladesh; 4 Department of Management and Engineering, Linköping University, 581 83 Linköping, Sweden

**Keywords:** Output, Energy use, CO_2_ emission, Trade, Middle-income countries, Q13, Q20, Q56

## Abstract

Middle-income countries are currently undergoing massive structural changes towards more industrialized economies. In this paper, we carefully examine the impact of these transformations on the environmental quality of middle-income countries. Specifically, we examine the role of sector value addition to GDP on CO_2_ emission nexus for middle-income economies controlling for the effects of population growth, energy use, and trade openness. Using recently developed panel methods that consider cross-sectional dependence and allow for heterogeneous slope coefficients, we show that energy use and growth of industrial and service sectors positively explain CO_2_ emissions in middle-income economies. We also find that population growth is insignificantly associated with CO_2_ emission. Hence, our paper provides a solid ground for developing a sustainable and pro-growth policy for middle-income countries.

## Introduction

The 2013 assessment report by the Intergovernmental Panel on Climate Change suggests that the largest contribution to total radioactive forcing (RF) in the world came from an increase in the atmospheric concentration of carbon dioxide (CO_2_) emissions since 1750. CO_2_ emissions are responsible for the 58.8% of the global greenhouse gasses (GHGs) (*The Little Green Data Book 2007*, World Bank). Without further effective policies to combat climate change, the OECD (2008) estimates the growth of GHG emissions of about 52% by 2050.

To the extent that energy consumption is the main source of carbon emissions, the essential question for every country is then how to promote economic growth without degrading environmental quality. Prior literature examine the causal interactions between energy consumption, carbon emissions, and overall economic growth for a number of groups of countries across regions, e.g., Pao and Tsai (2010) for BRIC countries; Arouri et al. (2012) for MENA countries; Borhan et al. (2012) for eight Asian countries; Moomaw and Unruh (1997) for 16 developed countries; Piaggio and Padilla (2012) for OECD countries; Coondoo and Dinda (2008) for a handful number of African, Asian, American, and European countries; and Hossain (2011) on newly industrialized countries. However, empirical literature on the sectoral growth effect on carbon emission is limited.

We argue that an exhaustive study on the sectoral growth effect on carbon emission involving the middle-income countries merits investigation for several reasons. *First*, over the last three decades, the economic significance of middle-income countries is growing in global growth paradigm. In the past three decades, these countries have been enjoying higher economic growth by transforming their economies from the primary agricultural sector to the energy-led industrial sector. Table 1 clearly demonstrates that on average, middle-income countries account for 14.84, 15.95, and 19.56% of the world share of GDP during the decades of 1980–1990, 1990–2000, and 2000–2010, respectively. This is an unprecedented 31.71% increase in growth from 1980 to 2010 in the world share of GDP.

**Table 1:** Average share of middle-income countries in GDP, sectoral GDP, energy use, emission, and population in respect to the world

Variables	Middle-income countries			Upper middle-income countries			Lower middle-income countries		
	1980–1990	1990–2000	2000–2010	1980–1990	1990–2000	2000–2010	1980–1990	1990–2000	2000–2010
GDP % of world	14.85	15.96	19.56	11.32	12.22	15.03	3.54	3.73	4.53
Industrial GDP (% of world)	17.16	20.38	27.02	13.39	16.04	21.66	3.70	4.34	5.36
Service GDP (% of world)	11.26	12.18	14.97	8.98	9.57	11.59	2.29	2.60	3.38
Agriculture GDP (% of world)	52.37	56.17	59.66	32.09	34.52	36.37	20.30	21.65	23.29
Energy use (% of world)	32.44	35.79	42.01	21.58	23.90	29.42	10.78	11.91	12.61
CO_2_ emission (% of world)	29.59	35.91	43.39	21.79	26.20	29.42	7.47	9.70	10.69
Population (% of world)	68.10	69.23	69.58	36.31	35.83	34.81	31.79	33.44	34.77

Source: World Bank (2013)

To fuel continued economic growth, today, middle-income countries alone consume about 42% of the world’s energy, indicating a 30% increase during the period of 1990–2010 and emitting 43.38% of the world’s total CO_2_ emissions, almost a 50% increase during the period of 1990–2010. Today, middle-income countries’ shares of the world GDP, energy use, and CO_2_ emission are 19.56, 43.01, and 43.39%, respectively, clearly indicating that an exhaustive study on the dynamic linkage of sectoral GDP, energy consumption, and CO_2_ emission is a serious academic and policy requirement, which earlier studies have overlooked. Furthermore, such investigation becomes even more interesting since almost 70% of the world’s population lives in middle-income countries.

**Fig. 1:**
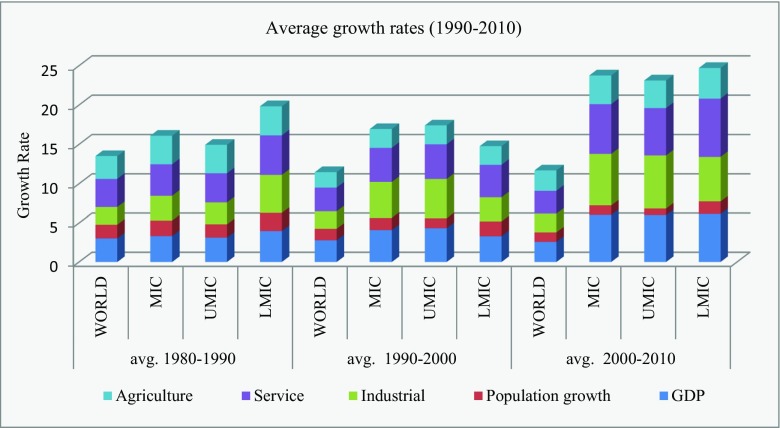
A comparison of the average growth of agricultural, service, and industrial sectors across the world and the middle-income countries (1990–2010). *MIC* middle-income countries, *UMIC* upper middle-income countries, *LMIC* lower middle-income countries (source: World Bank 2013)

*Second*, there is a significant structural difference in the economic growth achieved and pursued by countries across the world. World Bank (2010) suggests that, in the post-industrialized period, there is a tremendous growth in service output. The agriculture sector contributes only 2%, while the service sector contributes 66% of a high-income country’s share of GDP. In a disaggregate level, though the economic structure of middle-income countries is still dominated by agriculture—with output constituting 52.37, 56.17, and 59.66% for the decades of 1980–1990, 1990–2000, and 2000–2010, respectively, (see Fig. 1)—there is a stupendous level of growth achieved by middle-income countries in industrial and service sectors. Over the last three decades, the middle-income countries’ share of the world’s industrial output has been 17.16, 20.38, and 27.02%, respectively, indicating an average growth rate of 57.45%, and the middle-income countries’ share of the world’s industrial output has been 11.26, 12.18, and 14.97%, respectively, indicating an average growth rate of 33.01% over the same period. Among the middle-income countries, with respect to the world share of sectoral GDP, the upper middle-income countries enjoy superiority over lower middle-income countries in respect to industrial output, while the lower middle-income countries enjoy superiority over upper middle-income countries in respect to service output. These results clearly highlight the fast-changing structural transition of the economies of middle-income countries towards industrialization and the service sector. Therefore, the potential that these sectors are contributing differently to the CO_2_ emission level cannot be ruled out. However, empirical investigations on the relative contribution of sectoral GDP on CO_2_ emissions across regions are non-existent in this field. Though a recent study by Al Mamun et al. (2014) have addressed such concerns, their study did not consider the possibility of cross-sectional dependence in both output growth and CO_2_ emission. Moreover, their study ignored an important variable energy consumption. As mentioned earlier, since the 1990s, the global share of middle-income countries’ output in the agriculture sector has increased by 13.92% while in the industrial and service sectors, such growth has been 57.45 and 32.94%, respectively. Such an unparalleled and tangible economic transformation in middle-income countries might offer a new explanation on the output emission nexus. An empirical validation about the difference in the sector-wise contribution to CO_2_ emission within a cross-sectional dependence framework will contribute to developing an environmentally harmonious and properly blended pro-growth strategy for middle-income countries.

*Third*, achieving economic growth is always a political mandate that every government across the world wants to pursue. However, for middle-income countries, such a mandate is more pronounced than in other countries. This is because most middle-income countries are heavily populated (almost 70% of the world’s population lives in middle-income countries), and their governments are relatively more burdened and pressed to increase per capita income, provide employment (youth unemployment rate is 21% (Cho et al. 2012) in middle-income countries), and increase the standard of living for their citizens. What is the consequence of such political mandate? Studies suggest that over next three decades, some three billion people are expected to join a new global middle class, increasing the daily energy consumption. This unprecedented increase in global energy consumption will spur additional CO_2_ emissions. Studies such as those of Faiers et al. (2007) and Mills and Schleich (2012) suggest that technological sophistication, residential energy-efficient technology adoption, energy conservation, knowledge, and attitude towards energy savings are important steps in minimizing the negative effect of increasing energy use and economic growth. Arguably, middle-income countries lack such technological sophistication and have a weak infrastructure in terms of public awareness, regulations, and technology to promote low carbon and sustainable economic growth compared to high-income countries (Yanikkaya 2003). Therefore, an aggressive low-cost, pro-growth approach by middle-income countries that are not concerned with the environmental consequences of their output growth is an alarming reality. A study on the nature and causes of their shares of CO_2_ emission in the global atmosphere will enable appropriate policy formulation for the harmonious coexistence between economic growth and ecological balance.

*Fourth*, sociological research on the climate change science and climate policy has put attention on human dimensions including deforestation, industrial water pollution, ecological consequences (e.g., public health), greenhouse gas emissions, and sustainable development. The environmental sociology (Schnaiberg 1980) theory explains the complexity between the market liberalization and the environment sustainability, while the ecological modernization theory (e.g., Mol 1997) argues that the advanced market societies will improve resource efficiency through social and technological innovations. Previous research conducted by sociologists indicates that the national-level greenhouse gas emissions provides evidence that population size is a primary anthropogenic driver of total carbon emissions (e.g., Rosa et al. 2004; York et al. 2003; Rosa and Dietz 2012) and that globalization increases per capita emissions in lower-income nations (e.g., Jorgenson and Clark 2012). Industrialization and liberalization are two important drivers of global climate change (Rockström et al. 2009). They conclude that the rise of industrialization led to the use of fossil fuels and the power of industrial ignition to the production of commodities for expanding market exchange and capital accumulation (Foster et al. 2010).

*Finally*, a study on middle-income country’s sample has additional merits as well. It is well known that CO_2_ emission is a global phenomenon, and there is a vertical and horizontal channel for the atmospheric concentration of CO_2_ at least in a particular region. Therefore, it is possible that CO_2_ emissions in one country can affect another country. For example, the Indonesian forest fires in 1997 and 2013 had a severe effect on the emission level of Malaysia as well as Singapore. Thus, most of the earlier empirics to date in this field have serious methodological limitations. The methodological limitations stem not only from the inherent nature of the methodology applied but also from improperly contextualizing the problem addressed. CO_2_ emissions are a global problem, and a country-specific study cannot fully uncover the dynamic nexus between emissions and output, since in the age of globalization and trade liberalization, most of the today’s middle-income countries including China, India, Brazil, Malaysia, Indonesia, Turkey, and South Africa have adapted an export-oriented pro-growth strategy. A spur of foreign capital by multinational corporations (MNCs), combined with middle-income countries’ resources, is taking global productivity to new heights. The economic power of Indian and China in the global context clearly reaffirms such reality. Today, these middle-income countries are fiercely competing against each another in the international marketplace. Thus, the rise of output growth in these countries is cross-sectionally dependent. Alternatively, CO_2_ emissions resulted from output growth in one middle-income country can affect the size and intensity of the CO_2_ emission in another middle-income country.

Hence, quite candidly, a focus on only middle-income countries has the same problem. However, we argue that such problem in the selection of middle-income countries is not as serious since other left-out regions such as high-income countries are relatively far better equipped than middle-income countries to deal with CO_2_ emissions; at the same time, the low-income countries contribute so insignificantly to the global share of GDP that CO_2_ emission from their output growth might be ignored. Therefore, acknowledging the idea of cross-sectional dependence in the CO_2_ emission, the earlier literature focusing on a specific country can be criticized from the wrong contextualization of the CO_2_ emission nature, and literature focusing on specific regions (see Table 2) can be criticized for ignoring the possible effect of cross-sectional dependence in their estimation.

**Table 2:** Empirics on output and CO_2_ emission nexus focusing different regions

Authors	Data period	Region (countries)	Primary variable	Others controls	Methods	Key findings
Niu et al. (2011)	1971–2005	8 Asia-Pacific countries	GDP and CO_2_	Oil, coal, gas, electricity	Panel VECM-based Granger causality	GDP − CO_2_ ↑ CO_2_→GDP ↑ EU→CO_2_ ↑
Chiu and Chang (2009)	1996–2005	OECD	GDP and CO_2_	CPI	Panel threshold regression	GDP→CO_2_ ↑ CPI − CO_2_ ↓
Wang (2012)	1971–2007	98 countries	GDP and CO2		Dynamic panel threshold regression	GDP→CO_2_ ↑↓
Hocaoglu and Karanfil (2011)	1970–2008	G-7	CO_2_ and industrial value added in GDP		Hidden Markov models	Industrial GDP→CO_2_ ↑
Pao and Tsai (2010)	1992–2007	BRIC	CO_2_ and industrial value added in GDP	Energy use, FDI	Multivariate Granger causality	GDP ∩ CO_2_ EU→CO_2_ ↑ FDI − CO_2_
Al-mulali (2012)	1990–2009	Middle Eastern	CO_2_ and industrial value added in GDP	Energy use, FDI, trade	Pedroni cointegration, fully modified OLS, panel Granger causality test results	GDP, EU, FDI, trade→CO_2_ ↑
Coondoo and Dinda (2002)	1950–1992	World	CO_2_ and industrial value added in GDP		Panel Granger causality	GDP ↔ CO_2_
Lean and Smyth (2010)	1980–2006	ASEAN	CO_2_ and industrial value added in GDP	Energy use	Panel cointegration and Granger causality	EU ↔ CO_2_ ↑ GDP→CO_2_ ↑
Sohag et al. (2015)	1985–2012	Malaysia	Energy use and GDP per capita	Technology	ARDL technique	Technology→EU ↓ GDPC→EU ↑
Salahuddin and Gow (2014)	1980–2012	GCC	Energy use and GDP per capita	Energy use	Pooled mean group	GDP − CO_2_ no relation
Kivyiro and Arminen (2014)	1971–2009	Sub-Saharan Africa	Energy use and GDP per capita	Energy use, FDI	ARDL technique	GDP→CO_2_ ↑ EU→CO_2_ ↑

*ARDL* autoregressive distributed lag

## Methods

### Data description

We use the World Development Indicators (WDI) dataset from 1980 to 2012. We followed the World Bank classification (http://data.worldbank.org/about/country-classifications) of countries based on per capita income. There are five major classification groups, and we considered middle-income countries as our sample. There are two types of middle-income countries: lower middle-income countries (LMICs) and upper middle-income countries (UMICs), and we considered both groups in this study. Our dependent variable is CO_2_ emission per capita in metric tons. This includes CO_2_ produced during consumption of gas flaring and solid and liquid fuels. Other variables of the study include agriculture GDP, industrial GDP, and service sector value addition to GDP normalized by GDP. This will allow us to consider the relative impact of sector-wise decomposed GDP on CO_2_ emissions. Moreover, we consider population growth (PG), energy use (EU), and trade openness (TO) as other controls following earlier empirics in this area such as Cropper and Griffiths (1994), Lean and Smyth (2010), Pao and Tsai (2010), Begum et al. (2015), and Al-mulali (2012).

### Cross-sectional dependence in panel

In the wake of financial and trade liberalization, middle-income countries virtually followed a homogenous pattern of sectoral restructuring of their respective economies in their pursuit for achieving growth and self-sufficiency. Moreover, CO_2_ emissions are magnified by vertical and horizontal atmospheric channels. Hence, the cross-sectional dependence in error processes is likely since cross-correlation occurs frequently due to spatial spillover, omitted common factors, and interactions within the socioeconomic network (Pesaran and Tosetti 2011). Technically, when residual of one cross-sectional unit is influenced by another cross section, the standard panel methods provide biased estimators (Pesaran 2004). Hence, we employ the cross-sectional dependence (CD) test developed by Pesaran (2004) to investigate the possibility of the existence of contemporaneous correlation across countries. Unfortunately, such a contemporaneous correlation effect has been overlooked in the literature of CO_2_ emission as well as economic growth nexus (Al Mamun et al. 2014; Niu et al. 2011; Chiu and Chang 2009). Moreover, the presence of CD also compromises the findings of mean group, pooled mean group, and generalized methods of moments.

The null hypothesis of the CD test is cross-sectional independence. Specifically, the test follows the equation: $\mathrm{CD}={\left(\frac{TN\left( N-1\right)}{2}\right)}^{1/2}\overline{\widehat{P}}$, where *N* and *T* indicate the cross section and time dimensions, respectively, and $\overline{\widehat{\rho}}=\left(\frac{2}{N\left( N-1\right)}\right){\sum}_{i=1}^{N-1}{\sum_{j= i+1}^N\widehat{\rho}}_{i j}$, where ${\widehat{\rho}}_{ij}$ indicates the pair-wise, cross-sectional correlation coefficient of the residuals from the augmented Dickey-Fuller (ADF) regression. Next, we conduct the cross-sectionally augmented panel unit root test (CIPS) test following Pesaran (2007) using the equation: $\Delta {y}_{i t}={\alpha}_i+{K}_i t+{\beta}_i{y}_{i t-1}+{\gamma}_i{\overline{y}}_{t-1}+{\phi}_i\Delta {\overline{y}}_t+{\varepsilon}_{i t}$, where *t* =1,…, *T* and *i* = 1,…, *N*. In the equation, ${\overset{-}{y}}_t$ indicates the cross-sectional mean of *y*_*it*_, which is derived from ${\overset{-}{y}}_t={N}^{-1}\sum_{i=1}^N{y}_{i t}$. This test allows us to account for the contemporaneous correlation among *y*_*it*_. The null hypothesis of the test is *H*_0_ : *β*_*i*_ = 0 for all *i* and alternative hypothesis *H*_*a*_ : *β*_*i*_ < 0 for some *i*. Pesaran (2007) presents the test statistics as follows: $\mathrm{CIPS}\left( N, T\right)={N}^{-1}\sum_{i=1}^N{t}_i\left( N, T\right).$

### The model

The structure of our dataset and the contextual viewpoint of our research question necessitate the use of cross-correlated effect mean group (CCEMG) and augmented mean group (AMG) estimators developed by Pesaran (2006) and Eberhardt and Teal (2010), respectively. We also relax the assumption of CD and apply the mean group estimator developed by Pesaran and Smith (1995) to contrast our findings under CCEMG and AMG. The superiority of CCEMG and AMG over other estimators such as seemingly unrelated regression equations (SUREs) estimated under a generalized least square (GLS) technique that can address CD bias is quite appealing. Pesaran (2006) posits that SURE is not applicable for *N* > 10 and small time dimension (*T*). Moreover, SURE is a time-invariant estimator and the proposal of Ahn et al. (2001) to overcome this problem does not eliminates the entire set of concerns including the fact the error term may not be identically and independently distributed. In contrast, the CCEMG is efficient in the presence of unobserved common effects (Pesaran 2006) and it is asymptotically unbiased as both *N* and *T*→∞.

Hence, we estimate the following main model using CCEMG and AMG estimates.

1 $$ {\mathrm{lnCO}}_2={a}_j+{d}_j t+{\beta}_{j1}{\mathrm{lnGDPC}}_{j t}+{\beta}_{j2}{\mathrm{TO}}_{j t}+{\beta}_{j3} \ln {\mathrm{EU}}_{j t}+{\beta}_{j4}{\mathrm{PG}}_{j t}+{\varepsilon}_{j t\dots } $$

In the above equation, *j* stands for the cross-sectional dimension *j* = 1,…, *J* and period *t* = 1,…, *T*. We also estimate Eq. (2) by removing the GDP per capita and using the decomposed GDP contributed by various sectors to understand the dynamic differences among the contribution of the various sectors in CO_2_ emissions:

2 $$ {\mathrm{lnCO}}_2={a}_j+{d}_j t+{\beta}_{j1}{\mathrm{AGDP}}_{j t}+{\beta}_{j2}{\mathrm{IGDP}}_{j t}+{\beta}_{j3}{\mathrm{SGDP}}_{j t}+{\beta}_{j4}{\mathrm{TO}}_{j t}+{\beta}_{j4} \ln {\mathrm{EU}}_{j t}+{\beta}_{j5}{\mathrm{PG}}_{j t}+{\varepsilon}_{j t} $$

In the above equation, *a*_*j*_ is the country-specific effects and *d*_*j*_ *t* represents the heterogeneous country-specific deterministic trends. Note that *a*_*j*_ is related with the coefficient of all respective independent variables as follows: ${\beta}_{j1}=\frac{\alpha_{j1}}{1-{\alpha}_{j1}}$, ${\beta}_{j2}=\frac{\alpha_{j2}}{1-{\alpha}_{j1}}$, ${\beta}_{j3}=\frac{\alpha_{j3}}{1-{\alpha}_{j1}}$, ${\beta}_{j4}=\frac{\alpha_{j4}}{1-{\alpha}_{j1}}$, and ${\beta}_{j5}=\frac{\alpha_{j5}}{1-{\alpha}_{j1}}$.

It is important to note that we do not impose homogenous restrictions in the per capita GDP, sector value addition to GDP, trade openness, population growth, and energy consumption across the sample countries in estimating Eqs. (1) and (2). We consider the parameter vector of the slope coefficient *β*_*j*_ = (*β*_*j*1_,  *β*_*j*2_,  *β*_*j*3_,  *β*_*j*4_,  *β*_*j*5_) as heterogeneous across *N*. We also consider *u*_*jt*_ that follows ${u}_{j t={\overset{\acute{\mkern6mu}}{\tau}}_j{f}_t+{\varepsilon}_{j t}}$ and represent the short-run dynamic adjustments towards long-run equilibrium. The *f*_*t*_ is the vector of unobserved common shocks. Although *f*_*t*_ can be either stationary or non-stationary, it does not influence the validity of the estimates of CCEMG (Kapetanios et al. 2011). The parameters of CCEMG model are *β*_*j*_ = *β* + *ω*_*j*_ and represent the common parameter *β* across *N* while *ω*_*j*_ ∼ *IID*(0, *V*_*ω*_) (Pesaran 2006). The estimator of CCEMG is shown as follows: ${\widehat{\beta}}_{\mathrm{CCEMG}}={J}^{-1}\sum_{i=1}^J{\widehat{\beta}}_j$. We also use the AMG proposed by Eberhardt and Teal (2010) that follows that the first-difference ordinary least squares of pooled data and augmented with year dummies also capture the unobserved common effect among the cross-sectional units. The AMG also allows a group-specific estimator using the sample average of cross-sectional units.

## Results

In this study, we consider the impact of sectoral GDP normalized by GDP and energy use on CO_2_ emissions in middle-income countries. In order to estimate the model, we examined the possible cross-sectional dependence across countries in the panel for respective series (CO_2_ emission, GDP per capita, agriculture GDP, industrial GDP, service sector value addition to GDP, population growth, energy use per capita, and trade openness) by using the CD (Pesaran 2004) test. The results reported in Table 3 show that the null hypothesis of no contemporaneous correlation among estimated residuals is rejected for CO_2_ emission, GDP per capita, agriculture GDP, industrial GDP, service sector value addition to GDP, population growth, energy use per capita, and trade openness. Due to the presence of cross-sectional dependence, the panel unit root test proposed by Pesaran (2007) is applied.

**Table 3:** Cross-sectional dependence and unit root test

Variables	$\widehat{\rho}$	CD	CIPS^a^ (levels)	CIPS (first differences)
CO_2_	0.548	47.60^a^	5.699	13.035^a^
GDPC	0.624	151.34^a^	−1.501	−2.677^a^
AGDP	0.603	163^a^	1.228	−5.804^a^
IGDP	0.186	11.11^a^	2.883	−3.680^a^
SGDP	0.495	80.11^a^	0.551	−2.758^a^
PG	0.525	98.85^a^	−0.506	−2.546^a^
EU	0.562	38.27^a^	−1.550	−2.592^a^
TO	0.427	34.56	−1.087	−12.007^a^

$\widehat{\rho}$ is the average of correlation coefficients across all pairs, and CD denotes cross-sectional dependence test statistics. The model used to test the unit root hypothesis is the one with an intercept and trend. The CIPS test for panel unit root statistics developed by Pesaran (2007). The theoretical value of the CIPS statistic is given in Table II (C) of Pesaran (2007). Lowercase letters a, b, and c indicate the significance level at the 1, 5, and 10%, respectively

^a^CIPS runs the *t* test for unit roots in heterogeneous panels with cross-sectional dependence, proposed by Pesaran (2007)

It is important to examine the order of integration of the variables, as the asymptotic distribution of parameters depends on whether variables of interests are all *I*(1) or *I*(0) (see for details Wu et al. 2010). However, the result shows that the CIPS test accepts the null hypothesis of a unit root for all variables at a conventional level, while the CIPS test rejects the null of unit root when all the variables are first differenced.

This study examines the long-run effects of per capita GDP, population growth, and energy use on CO_2_ emission in the context of 83 middle-income countries. Initially, we consider the standard panel econometrics approach of panel data analysis, e.g., fixed effect (FE), random effect (RE), fixed effect instrumental variable (FE-IV), and fixed effect first difference (FE-FD). We apply the statistical approaches to analyze our model to examine its validity by applying the CD and CIPS tests on the residuals. This is fundamentally important for the panel data analysis because the validity of an obtained result from any panel estimator depends on the two important diagnostic tests: cross-sectional dependence and unit root test since the residuals of the model should be cross-sectionally independent and stationary (see for details Sadorsky 2013). In order to check the robustness of the estimation procedure, we apply the estimation for subsample of upper middle-income countries and lower-middle-income countries to examine the extent the finding changes with the income level.

The empirical results of the models, estimated by using pooled ordinary least squares (POLS), FE, FE-IV, and FE-FD estimators, are presented in Table 4. The results from the last two rows of Table 4 indicate that the CD test rejects the null hypothesis of cross-sectional independence of residuals for all four estimators: POLS, FE, FE-IV, and FE-FD. Moreover, the null hypothesis is that the presence of unit root is accepted by the CIPS test for all four estimators except the FE-FD estimator in the context of lower middle-income countries. The results do not vary in the case of clustered sample countries. The cross-sectional dependency and unit root in the residual of all statistical models indicate a poor model fit. Therefore, these preliminary results signal that only the dynamic models should be considered.

**Table 4:** The impact of GDP per capita on CO_2_ emission per capita: statistical analysis (1980–2012) for the full sample and clustered sample countries

	All middle-income countries				Upper middle-income country				Lower middle-income country			
	DV/CO_2_											
	POLS	FE	FE-IV	FE-FD	POLS	FE	FE-IV	FE-FD	POLS	FE	FE-IV	FE-FD
PG	−0.062^a^	0.041^c^	−0.360	0.003	−0.118^a^	0.020	−0.052	−0.025	−0.117^a^	0.0456^c^	0.000	0.0833^a^
SE	−0.024	−0.024	−0.267	−0.018	−0.030	−0.039	−0.049	−0.026	−0.032	−0.024	−0.045	−0.023
TO	0.000	−0.010^a^	0.020	0.002^a^	0.001	−0.005^a^	0.000	0.002^b^	−0.004^a^	−0.0145^a^	−0.0184^a^	0.001^b^
SE	−0.001	−0.001	−0.019	−0.001	−0.001	−0.001	−0.003	−0.001	−0.001	−0.001	−0.001	−0.001
LEU	2.447^a^	1.630^a^	9.371^b^	0.811^a^	3.407^a^	2.072^a^	2.666^a^	0.954^a^	1.544^a^	0.971^a^	−2.313^a^	0.578^a^
SE	−0.040	−0.074	−4.759	−0.081	−0.056	−0.110	−0.248	−0.130	−0.053	−0.089	−0.498	−0.081
LGDPC	0.125^a^	0.671^a^	−14.670	0.573^a^	−0.039	0.730^a^	−0.922	0.708^a^	0.317^a^	0.717^a^	5.419^a^	0.262^b^
SE	−0.042	−0.075	−9.419	−0.109	−0.064	−0.113	−0.621	−0.167	−0.073	−0.089	−0.694	−0.118
Constant	−14.44^a^	−12.55^a^	50.220	−0.005	−19.88^a^	−16.37^a^	−7.60^b^	0.004	−9.793^a^	−8.603^a^	−20.74^a^	−0.012^b^
SE	−0.338	−0.527	−38.570	−0.007	−0.610	−0.937	−3.385	−0.012	−0.597	−0.462	−1.937	−0.006
Observations	2586	2586	2581	2501	1353	1353	1351	1308	1233	1233	1230	1193
*R* ^2^	0.670	0.315		0.066	0.747	0.301		0.074	0.505	0.471		0.070
Number of country		82	82	82		43	43	43		39	39	39
CD	46.340^a^	36.880^a^	22.040^a^	12.540^a^	43.52^a^	56.43^a^	10.79^a^	22.08^a^	50.05^a^	24.61^a^	93.82^a^	17.61^a^
CIPS	1.778	1.990	0.915	−8.088^a^	1.778	1.990	0.915	−8.088^a^	1.473	0.541	1.074	−7.787^a^

The estimation is from a balanced panel of 82 middle-income countries covering the period of 1980–2012. The superscripts a, b, and c denote significance at the 1, 5, and 10% levels, respectively. SE indicates standard error of the estimates

*POLS* pooled OLS, *FE* fixed effect, *FE-IV* fixed effect instrumental variables, *FE-FD* fixed effect first difference

The results from dynamic estimators like the mean group (MG), CCEMG, and AMG are presented in Table 5. Since the CD and CIPS tests reject the null hypothesis of cross-sectional dependence and unit root, respectively, the residuals obtained the dynamic estimator, except MG (second last row for the second column of Table 5). These findings clearly indicate the goodness of fit of the models.

**Table 5:** The impact of GDP per capita on CO_2_ emissions per capita (1980–2012) for the full sample and clustered sample countries

	All middle-income countries			Upper middle-income country			Lower middle-income country		
	MG	CCEMG	AMG	MG	CCEMG	AMG	MG	CCEMG	AMG
Population growth	0.123 (0.077)	0.130 (0.086)	0.054 (0.064)	0.163 (0.123)	0.155 (0.127)	0.142 (0.126)	0.0783 (0.092)	0.0952 (0.112)	0.109 (0.084)
Trade openness	−0.002 (0.001)	−4.060 (0.001)	−0.001 (0.001)	−0.004 (0.002)	−0.001 (0.002)	−0.004^c^ (0.002)	0.001 (0.001)	0.001 (0.001)	0.001 (0.001)
Energy use (per capita)	2.048^a^ (0.364)	1.795^a^ (0.309)	1.942^a^ (0.366)	2.667^a^ (0.567)	2.300^a^ (0.426)	2.481^a^ (0.537)	1.365^a^ (0.424)	1.184^a^ (0.368)	1.079^a^ (0.319)
GDP (per capita)	0.496^a^ (0.168)	0.089 (0.455)	0.439^b^ (0.196)	0.738^b^ (0.300)	0.269 (0.691)	0.386 (0.382)	0.229^b^ (0.116)	0.169 (0.270)	0.238^b^ (0.111)
Common dynamic process			0.818^a^ (0.270)			1.100^b^ (0.429)			0.101 (0.214)
Constant	−16.12^a^ (2.797)	−8.891^c^ (4.903)	−14.49^a^ (2.865)	−21.85^a^ (4.562)	−17.86^c^ (9.577)	−17.50^a^ (5.215)	−9.797^a^ (2.772)	−6.266^b^ (2.799)	−8.010^a^ (1.979)
Observations	2586	2586	2586	1353	1353	1353	1233	1233	1233
Number of country	82	82	82	43	43	43	39	39	39
CD	2.30^b^	0.82	−0.25	1.46	0.74	−1.63	1.59	0.27	0.68
CIPS	14.088^a^	−22.137^a^	−16.486^a^	−11.266^a^	−15.275^a^	−12.719^a^	−8.799^a^	−17.030^a^	−9.469^a^

The estimation is from a balanced panel of 82 middle-income countries covering the period of 1980–2012. The superscripts a, b, and c denote significance at the 1, 5, and 10% levels, respectively. Standard error is within parentheses

*MG* mean group, *CCEMG* cross-correlated effect mean group, *AMG* augmented mean group

## Discussion

The concentration of greenhouse gasses in the atmosphere is increasing because of various human activities. Therefore, population growth is the core factor in explaining CO_2_ emission dynamics (Bongaarts 1992) in middle-income countries. There is a common belief that population growth has been fostering greenhouse gas emissions by burning energy, urbanization, deforestation, and so on (Kerr and Mellon 2012; Meyerson 1998). However, as long as the production theory is a concern, where capital and labor are substitutes for each other, replacement of human labor for capital may reduce the burning of pollutant energy, hence lower CO_2_ emission. Given that the population growth rate in developed economies is lower than in the least developing countries (LDCs) (Bongaarts 1992), the slightly higher population growth in middle-income countries, when compared to high-income countries, cannot be considered as the primary driver for CO_2_ emission. The finding of this study shows a similar result, as the coefficient of population growth is positive but insignificant. The result is consistent throughout the three dynamic estimators for both full and clustered samples. Hence, the distribution of energy use, rather than population growth, is the prime catalyst of CO_2_ emission.

In an era of globalization, it has been a central focus whether cross-border integration helps or hurts the health of the environment. The trade theory of Helpman and Krugman (1985) explains that trade openness promotes physical output while numerous empirics suggest increased output is positively associated with CO_2_ emission. Thus, trade openness might lead to higher CO_2_ emission. However, the equation is not so simple and straight forward. In this context, Ang (2009) argued that trade openness promotes higher productivity for resources including energy, which might lead to diminishing marginal emission from using energy when compared to the output growth.

Furthermore, Yanikkaya (2003) stated that due to the trade openness, technologies have become readily available in a country from trading countries. Therefore, economic efficiency and better technology would promote the quality of economic growth, i.e., less negative externalities. The estimated results under the AMG estimator reported in Table 3 suggest that such an idea is valid in the case of upper middle-income countries. The result posits that there are other controlling factors, as a 1-unit increase in openness would lead to a 0.003-unit reduction of CO_2_ emission. In the case of full sample countries and lower middle-income countries, the impact of trade openness is inconclusive. This finding is also consistent with the existing literature, e.g., Frankel and Rose (2005), for 38 countries ranging from high democracy to low democracy; Shahbaz et al. (2013a) for Indonesia; Shahbaz et al. (2013b) for South Africa; and Shahbaz et al. (2014) for low-, middle-, and high-income countries.

Regarding the relation between energy consumption and CO_2_ emission, there is a little crookedness in empirical studies though there are differences in the country-specific long-run elasticity across the sample due to the differences in the level of technological advancement. In the case of middle-income countries, results confirm a positive and statistically significant parameter of energy use per capita, which indicates that it intensifies the CO_2_ emission level. The finding is consistent across the board under all estimators. In comparison with the other factors in the model, the elasticity of CO_2_ emission with respect to energy consumption is disproportionately high under all the three estimators. Moreover, the coefficient is higher in upper middle-income countries compared to low-middle-income countries. A possible explanation for such result lies in the fact that upper middle-income countries are relatively more industrialized than lower middle-income countries. The finding is consistent with the literature, e.g., Shahbaz et al. (2014) in low-, middle-, and high-income countries; Hossain (2011) in newly industrialized countries (Brazil, China, India, Malaysia, Mexico, Philippines, South Africa, Thailand, Turkey); Ozturk and Acaravci (2010) in Turkey; Lotfalipour et al. (2010) in Iran; and Ang (2007) in Malaysia.

Regarding per capita income, the relation with CO_2_ emissions largely depends on three important mechanisms (Brock and Taylor 2005): the scale of production, composition or means of production, and technology used in the production process. Firstly, when the composition of output and technology are constant, CO_2_ emission increases along with the scale of economic activities. Secondly, for a fixed volume of economic output and given technology, emission would rise and fall depending upon dynamics of the composition, e.g., emission-intensive factors of production. Lastly, the intensity of emission or emission per unit of output would fall due to technological progress, holding the other things constant.

In respect to the aggregate effect of these three factors, the relation between economic growth and CO_2_ emission may become linear, U shape, inverted U shape, or any other shape (Wagner 2008). Although many previous studies confirmed the presence of an environmental Kuznets curve (EKC) in many economies around the world, the fact of the matter (quite unfortunately) is that absolute CO_2_ emission is rising globally. Our result also confirms that GDP per capita is a positive factor in CO_2_ emissions for all middle-income countries. This means that to thrive and to achieve further economic growth in middle-income countries, there must be serious thought about the impending negative effects of CO_2_ emission.

Finally, our attention is to address the relative contribution of different sector’s outputs on CO_2_ emissions. Table 6 presents the results. The result suggests that the coefficient of agriculture GDP is positive but statistically insignificant at the level of CO_2_ emission in all middle-income countries. Alternatively, the traditional sector of the economy is less responsible for the CO_2_ emissions compared to the sophisticated manufacturing and service sectors.

**Table 6:** The impact of decomposed GDP on CO_2_ emission (1980–2012) for the full sample and clustered sample countries

	All middle-income countries			Upper middle-income countries			Lower middle-income countries		
	MG	CCEMG	AMG	MG	CCEMG	AMG	MG	CCEMG	AMG
AGDP	0.0002 (0.002)	−0.0013 (0.003)	0.0012 (0.003)	−0.0013 (0.004)	−0.0012 (0.002)	−0.001 (0.005)	0.002 (0.002)	−0.0012 (0.003)	0.0023 (0.002)
IGDP	0.030^b^ (0.014)	0.031^c^ (0.017)	0.035^b^ (0.016)	0.052^b^ (0.0260)	0.045^b^ (0.023)	0.061^b^ (0.028)	0.005 (0.006)	0.0004 (0.006)	0.006 (0.006)
SGDP	0.027^c^ (0.015)	0.0225 (0.015)	0.0281^c^ (0.0147)	0.0510^c^ (0.0277)	0.038^c^ (0.021)	0.054^b^ (0.027)	−0.0004 (0.005)	−0.0052 (0.006)	−0.0005 (0.005)
PG	0.132 (0.085)	0.117 (0.082)	0.0707 (0.0793)	0.152 (0.143)	0.304^b^ (0.147)	0.133 (0.141)	0.109 (0.082)	0.0540 (0.076)	0.020 (0.063)
EU	2.228^a^ (0.335)	1.805^a^ (0.334)	2.251^a^ (0.414)	2.917^a^ (0.475)	2.57^a^ (0.583)	2.96^a^ (0.641)	1.450^a^ (0.444)	0.854^b^ (0.346)	1.449^a^ (0.501)
Common dynamic process			0.141 (0.399)			0.0535 (0.480)			0.242 (0.193)
Constant	−16.20^a^ (2.830)	−10.23^a^ (3.016)	−16.59^a^ (3.860)	−22.75^a^ (4.543)	−12.61 (8.115)	−23.58^a^ (6.561)	−8.799^a^ (2.776)	−3.872^b^ (1.802)	−8.580^a^ (3.064)
Observations	2599	2599	2599	1380	1380	1380	1219	1219	1219
No. of country	83	83	83	44	44	44	39	39	39
CD	1.85^c^	0.76	0.24	−0.04	0.56	−0.72	2.74^a^	1.06	1.43

The estimation is from a balanced panel of 82 middle-income countries covering the period of 1980–2012. The superscripts a, b, and c denote significance at the 1, 5, and 10% levels, respectively. Standard error is within parentheses

*MG* mean group, *CCEMG* cross-correlated effect mean group, *AMG* augmented mean group

A striking finding is that higher industrialization has led to a relatively higher level of CO_2_ emissions in all middle-income countries. The effect of industrial GDP is positive and significant for all middle-income and higher middle-income countries, but not for lower middle-income countries. The results reported in Table 6 show that the sophisticated service sector is responsible for intensifying CO_2_ emission levels across the middle-income countries. However, this finding is attributed for upper middle-income countries, not for the lower middle-income countries. Therefore, the overall effect of the sectoral GDP on CO_2_ emission is that the contribution of the industrial sector is more prominent than the service GDP.

## Conclusion and policy implications

We estimate the effect of economic growth, sectoral GDP, population growth, energy consumption, and trade openness on CO_2_ emission using the balanced panel data for middle-income countries from 1980 to 2012. The findings are important from the perspective of industrialized and developing countries.

The findings have overcome the problem of the cross-sectional bias in the data structure. Therefore, the estimates are a product of a more efficient and economic contextualization of the problem. Moreover, we have dealt with the sample of the most significant countries, i.e., middle-income countries driving the growth of world today. The most important variable that contributed to the growth in CO_2_ emission in middle-income countries has been identified as energy use. This is evident in both the upper and lower middle-income countries. This finding indicates that for the middle-income countries to reduce the CO_2_ emission, the efficiency in energy use should be given priority. In fact, the combined values of parameters of all other variables are much smaller than the beta of energy use in both models under all alternative estimates. In contrast to findings in the sociological literature (e.g., Rosa et al. 2004; York et al. 2003; Rosa and Dietz 2012), population growth was not significantly related to CO_2_ emissions. We found that distribution of energy use, rather than population growth, is the prime catalyst of CO_2_ emission. Future work should, however, evaluate this result carefully on country-specific cases to further illuminate the relationship between population growth and emissions. Finally, the role of agriculture GDP in CO_2_ emission could not be established, while industrial GDP is more responsible for CO_2_ emission than service GDP across middle-income countries. Therefore, the growing trend of industrialization in the middle-income countries should be planned in such a way that increases the energy efficiency of the production process, which can substantially reduce the level of CO_2_ emissions in the middle-income countries.

## Appendix

**Table 7:** List of the countries

Upper middle-income countries		Lower middle-income countries	
Albania	Iran, Islamic Rep.	Armenia	Nigeria
Algeria	Jamaica	Bhutan	Pakistan
Angola	Jordan	Bolivia	Paraguay
Argentina	Kazakhstan	Cameroon	Philippines
Azerbaijan	Lebanon	Cape Verde	Samoa
Belarus	Macedonia, FYR	Congo, Rep.	Senegal
Belize	Malaysia	Cote d’Ivoire	Sri Lanka
Bosnia and Herzegovina	Mexico	Djibouti	St. Vincent and the Grenadines
Botswana	Namibia	Egypt, Arab Rep.	Sudan
Brazil	Panama	El Salvador	Swaziland
Bulgaria	Peru	Georgia	Syrian Arab Republic
China	Romania	Ghana	Ukraine
Colombia	Seychelles	Guatemala	Uzbekistan
Costa Rica	South Africa	Guyana	Vanuatu
Cuba	St. Lucia	Honduras	Vietnam
Dominica	Suriname	India	Yemen, Rep.
Dominican Republic	Thailand	Indonesia	Zambia
Ecuador	Tonga	Kiribati	
Fiji	Tunisia	Moldova	
Gabon	Turkey	Mongolia	
Grenada	Turkmenistan	Morocco	
Hungary	Venezuela, RB	Nicaragua	
